# Systematic Analysis of Self-Reported Comorbidities in Large Cohort Studies – A Novel Stepwise Approach by Evaluation of Medication

**DOI:** 10.1371/journal.pone.0163408

**Published:** 2016-10-28

**Authors:** Tanja Lucke, Ronald Herrera, Margarethe Wacker, Rolf Holle, Frank Biertz, Dennis Nowak, Rudolf M. Huber, Sandra Söhler, Claus Vogelmeier, Joachim H. Ficker, Harald Mückter, Rudolf A. Jörres, COSYCONET-Consortium

**Affiliations:** 1 Institute and Outpatient Clinic for Occupational, Social and Environmental Medicine, University Hospital LMU Munich, München, Germany; 2 Comprehensive Pneumology Center Munich, DZL, German Center for Lung Research, München, Germany; 3 Center for International Health, Ludwig-Maximilian University Munich, München, Germany; 4 German Research Center for Environmental Health, Institute of Health Economics and Health Care Management, Member of the German Center for Lung Research, Comprehensive Pneumology Center Munich (CPC-M), Neuherberg, Germany; 5 Institute for Biostatistics, Hannover Medical School, Hannover, Germany; 6 Thoracic Oncology Center Munich (TOM), University Hospital LMU Munich, München, Germany; 7 Pulmonary and Critical Care Medicine, Department of Medicine, University Medical Centre Giessen and Marburg, Philipps-University, Marburg, Germany; 8 Department of Respiratory Medicine, Allergology and Sleep Medicine, Klinikum Nuremberg, Nürnberg, Germany; 9 Paracelsus Medical University Nuremberg, Nürnberg, Germany; 10 Walther-Straub-Institute for Pharmacology and Toxicology, Ludwig-Maximilian University Munich, München, Germany; Hunter College, UNITED STATES

## Abstract

**Objective:**

In large cohort studies comorbidities are usually self-reported by the patients. This way to collect health information only represents conditions known, memorized and openly reported by the patients. Several studies addressed the relationship between self-reported comorbidities and medical records or pharmacy data, but none of them provided a structured, documented method of evaluation. We thus developed a detailed procedure to compare self-reported comorbidities with information on comorbidities derived from medication inspection. This was applied to the data of the German COPD cohort COSYCONET.

**Methods:**

Approach I was based solely on ICD10-Codes for the diseases and the indications of medications. To overcome the limitations due to potential non-specificity of medications, Approach II was developed using more detailed information, such as ATC-Codes specific for one disease. The relationship between reported comorbidities and medication was expressed by a four-level concordance score.

**Results:**

Approaches I and II demonstrated that the patterns of concordance scores markedly differed between comorbidities in the COSYCONET data. On average, Approach I resulted in more than 50% concordance of all reported diseases to at least one medication. The more specific Approach II showed larger differences in the matching with medications, due to large differences in the disease-specificity of drugs. The highest concordance was achieved for diabetes and three combined cardiovascular disorders, while it was substantial for dyslipidemia and hyperuricemia, and low for asthma.

**Conclusion:**

Both approaches represent feasible strategies to confirm self-reported diagnoses via medication. Approach I covers a broad spectrum of diseases and medications but is limited regarding disease-specificity. Approach II uses the information from medications specific for a single disease and therefore can reach higher concordance scores. The strategies described in a detailed and reproducible manner are generally applicable in large studies and might be useful to extract as much information as possible from the available data.

## Introduction

Comorbidities in chronic conditions such as Chronic Obstructive Pulmonary Diseases (COPD) are known to influence prognosis, health status and therapy options [1]. For large cohorts the assessment of comorbidities is challenging and different tools are used to obtain and evaluate information on coexisting diseases (e.g. Charlson-Comorbidity-Index [2], ATS-DLD-78 [3]).

Self-reporting of diagnoses is a common approach to collect data on health status [4]. Much work has been invested to assess the value of self-reported data in comparison to other sources of information like pharmacy records on medications, or medical records from general practitioners (GP), nurses etc. [5–8]. Information on comorbidities given by the patients has been described to be reliable [9–11] especially for chronic conditions such as diabetes or heart disease [12]. Despite this, depending on the disease and several influencing factors it was found that in some cases patients’ reports tend to underestimate relevant comorbidities [5–7] but overestimation could also be demonstrated [12]. Therefore it has been recommended to take into account all available sources of information in the assessment of comorbidities [7, 13].

To identify and predict frailty in the elderly, Coelho et al. not only collected health data from self-reports but also evaluated specific medications [14]. Medication was categorized into groups of indications (e.g. cardiovascular, metabolic), however the categories were rather broad and no detailed description was given how to categorize and match the medications. The need for a combined evaluation of comorbidities and medication is also evident in the German COPD cohort COSYCONET which comprises more than 2500 patients [15–17]. In order to use as much information as possible we developed a novel categorization in which the concordance between medication and patients’ reports on comorbidities is evaluated. This approach which is described in all necessary detail might also be useful for other large cohort studies on respiratory or other diseases probing the influence of comorbidities on outcome and prognosis.

## Methods

COSYCONET is a multicenter cohort study focusing on disease progression over time in interaction with comorbidities [15]. All assessments were approved by the central (Marburg (Ethikkommission FB Medizin Marburg) and local ethical committees (see S1 Ethics Committees) and written informed consent was obtained from all patients. The study was in accordance with the declaration of Helsinki. The present analysis is based on data from the recruitment visit comprising COPD-patients of categories GOLD 0-IV [18] (n = 2653; for basic characteristics see Table 1). Data collection was performed from September 2010 to December 2013. All patients were required to be diagnosed with COPD or chronic bronchitis and an age of at least 40 years was required. Only few exclusion criteria were defined (e.g. lung surgery with great volume reduction, exacerbations within 4 weeks prior to baseline visit) since the study aimed at capturing a broad spectrum of patients with COPD. Comorbidities were assessed using a predefined list comprising a total of 51 (combined) diseases, as well as free text information. Moreover, patients were asked to bring all medication (original packages) to their study visit. Patients’ previous medical records were not evaluated due to issues of data quality and completeness. We solely used self-reports of physician-diagnosed conditions for morbidities, with medication brought to the visit being the supplementary source to be compared with diagnoses. Two approaches (I and II, see description below) were developed for a quality comparison. Approach II aimed at overcoming some of the limitations inherent to Approach I.

**Table 1:** Basic characteristics of the patients stratified according to COPD GOLD categories 0–4.

Patients (n)	Age Mean (±SD)	BMI Mean (±SD)	# of self-reported comorbidities Mean (±SD)	Male Total number (%)
All (2653)	65.0 (±8.6)	27.0 (±5.4)	6.0 (±3.4)	1575 (59.4)
GOLD 0 (362)	64.8 (± 9.7)	29.0 (± 5.8)	7.1 (± 3.7)	179 (49.4)
GOLD 1 (206)	66.2 (± 8.7)	26.6 (± 4.6)	6.2 (± 3.3)	124 (60.2)
GOLD 2 (962)	65.7 (± 8.5)	27.4 (± 5.1)	5.9 (± 3.3)	579 (60.2)
GOLD 3 (874)	65.0 (± 8.2)	26.4 (± 5.4)	5.8 (± 3.3)	533 (61.0)
GOLD 4 (249)	62.1 (± 7.9)	24.4 (± 5.0)	5.1 (± 3.0)	160 (64.3)

### Approach I for comparison of self-reported diagnoses

The first step was to transform comorbidities and medications into codes which could be matched by computer analysis. “Non-insulin dependent diabetes mellitus” and “insulin-dependent diabetes mellitus” were combined into “diabetes mellitus”, and reflux, gastritis and gastric and duodenal ulcerations into “gastro-intestinal disorders” (GI). In addition to the individual analyses, hypertension, coronary heart disease and heart failure were merged into a disease entity “combined cardiovascular disorder” to take account of the fact that there was a lack of medication specific for the single diseases, but specific medication for their combination is available. “Mental disorders” comprised depression, anxiety, panic attacks and psychotic disorders.

#### ICD10-Codes for comorbidities

To classify the reported diagnoses the system of International Classification of Diseases, version 10 (ICD10 [19]) was employed. ICD10-Codes comprise up to 5 digits (structure: X00.00). From the patients’ reports only limited information was available necessitating the definition of lumped categories. Thus, comorbidities were coded by ICD10-Codes but in most cases without applying subcategories. Except for sleep apnea, disturbed blood flow in the legs, and vein thrombosis we used only the first three digits.

In some instances a combination of three-digit codes was required since the single codes were not sufficient to describe the comorbidities. This applied e.g. for the integrative category of “coronary heart disease” which comprised I20: Angina pectoris, I24: Other acute ischemic heart diseases, and I25: Chronic ischemic heart disease. Table 2 shows all comorbidities mentioned by more than 400 patients or considered relevant in COPD. The full table of categories can be found in the S1 Table.

**Table 2:** ICD10 coding of relevant and/or prevalent comorbidities as used in the analysis of Approach I.

Disease (% prevalence self-reports)	Associated ICD10-Codes
Hypertension (56.1%)	any codes from I10 to I15
Dyslipidemia (39.0%)	E78
Gastrointestinal (36.5%)	any codes from K20 to K31 or R12
Mental disorders (21.4%)	F00 or any codes from F07 to F09 or any codes from F20 to F49 or any codes from F51 to F99
Asthma (18.5%)	J45 or J46
Hyperuricemia (16.6%)	E79 or M10
Coronary heart disease (16.1%)	I20 or I24 or I25
Osteoporosis (15.2%)	any codes from M80 to M85
Diabetes mellitus (13.6%)	any codes from E10 to E14
Combined cardiovascular disorder (60.8%)	any codes from I10 to I15; I20 or I24 or I25; I50

#### Transformation of ICD10-Codes of medication for the purpose of matching

Indications for each reported drug were retrieved from the AiDKlinik software program [20] using the PZN (pharmacological registration number in Germany) if available, or alternatively names, dosages and companies. In the next step a special dictionary was created to translate and re-code from the detailed 5-digit ICD10-Codes of the medication into single or multiple 3-digit-codes. This enabled the matching with the comorbidity codes. Table 3 illustrates the re-coding algorithm for selected comorbidities (for the full dictionary see S2 Table). Each code occurred only once for each patient even if more than one drug with the same indication had been prescribed. The conversion from detailed ICD10-Codes into the comprehensive, merged codes and their matching with comorbidity ICD10-Codes was performed in the programming language R 3.2.2 [21].

**Table 3:** List of ICD10-Codes used for comparison of comorbidities and medication.

Disease	Disease ICD10-Code	Leading three digits of medication ICD10-Codes
Hypertension	I10-I15	- any code from I10.XX to I15.XX
Dyslipidemia	E78	- E78.XX
Gastrointestinal (GI)	K20-K31; R12	- any code from K20.XX to K31.XX- R12.XX
Mental disorders	F00; F07-F09;F20-F49; F51-F99	- F00.XX- F07.XX or F08.XX or F09.XX- any code from F20.XX to F49.XX - any code from F51.XX to F99.XX
Asthma	J45-J46	- J45.XX or J46.XX
Hyperuricemia	E79; M10	- E79.XX- M10.XX
Coronary heart disease	I20; I24; I25	- I20.XX or I24.XX or I25.XX
Osteoporosis	M80-M85	- any code from M80.XX to M85.XX
Diabetes mellitus	E10-E14	- any code from E10.XX to E14.XX
Combined cardiovascular disorder	I10-I15; I20, I24, I25; I50	- any code from I10.XX to I15.XX- I20.XX or I24.XX or I25.XX- I50.XX

The second column of the table shows the ICD10-Codes of the diseases listed in the first column. The third column shows the merged ICD10-Codes of medication that were assigned to the ICD10-Codes of column 2. As illustrated in column 3, for the assignment the leading three digits of the medication ICD10-Codes were used.

#### Matching of ICD10-Codes between comorbidities and medication

The comparison led to three different classes. Either the intake of a medication was concordant with the reported disease (class 1), or a disease was reported without a matching medication (class 2). The possibility that there was a medication without a corresponding disease in the patient’s report was not evaluated as medications were not necessarily specific for a disease. The natural limitation of this approach results from the fact that medications were classified according to their indication in a given comorbidity but without requiring that this comorbidity was the only indication for this medication.

### Approach II for comparison of self-reported diagnoses

The basic idea of Approach II was to identify drugs having only one indication (according to the ICD10-Code indication list of the AiDKlinik database) and to use only these drugs to verify a reported disease. Such drugs could also serve as indicators of a disease if the comorbidity was not reported by the patient. This approach seemed reasonable especially for chronic conditions requiring sustained treatment.

#### Identification of medications specific for a disease

The 9 diseases and one disease combination chosen for analysis were hypertension, dyslipidemia, GI, mental disorders, asthma, coronary heart disease, hyperuricemia, osteoporosis, diabetes mellitus, as well as combined cardiovascular disorder. This combination was chosen not from a clinical but a pharmaceutical perspective, since many frequently prescribed medications (beta blockers, ACE inhibitors, AT_1_ receptor antagonists) are indicators of at least one of the conditions included in the combined cardiovascular disorder. In a first step the AiDKlinik database was searched for specific ATC-Codes indicating exclusively one disease (disease complex).

In the second step two qualified researchers (TL pharmacist, HM chemist and physician with focus on pharmacology) verified the specificity of medications. All drugs reported in COSYCONET were labeled with the respective ATC-Codes. The tables with all specific codes needed to categorize diseases can be found in the S3–S12 Tables. In most cases only drugs of the latest version of the Red List (= Rote Liste; a German registry of marketed drugs, including EU licenses) were included, with the exception of very few medications which were available at the time of the visit but out of trade at the time of the evaluation. The information on the drugs was used to create concordance scores for comorbidities by comparison with their specific medications.

#### Definition of concordance scores A and B

The first step was to match ATC-Codes specific for a disease with the diagnoses reported by the patient. Concordance score A was assigned if both were in accordance with each other. Patients taking at least one medication specific for a particular condition but not reporting this disease received score B.

### Comparison and Combination of Approaches I and II

The patients which could not be categorized into concordance scores A or B using specific medications could only be analyzed using the weaker information from Approach I. Approach II provides a sufficient condition for the presence of the disease, therefore patients can be considered with high confidence to have the respective comorbidity. In contrast in Approach I there was only medication compatible with the disease or no medication for the disease at all. The scores A and B from Approach II were kept in the combined approach. Patients with class 1 from Approach I (intake of a (nonspecific) drug plus report of a corresponding disease) were labeled as concordance score C, and patients with class 2 (report only) as concordance score D. The combined approach thus enabled the definition of four concordance scores which were computed for the comorbidities selected for analysis (see Tables 2 and 4). The matching procedures were again performed by special R-codes.

**Table 4:** Overview on the information used for defining the concordance scores.

Concordance score for analyses	Self-reported diagnosis	Specific medication	Non-specific medication
A	+	+	
B	-	+	
C	+	-	+
D	+	-	-

The (+) indicates the information that was used in assigning the concordance scores A-D. The (-) symbolizes the lack of information which therefore could not be used for the assignment. Please note that the scores C and D were only assigned to patients who were not already categorized in scores A and B. The empty boxes indicate information that was not considered according to the definition of the concordance scores.

## Results

**Fig 1:**
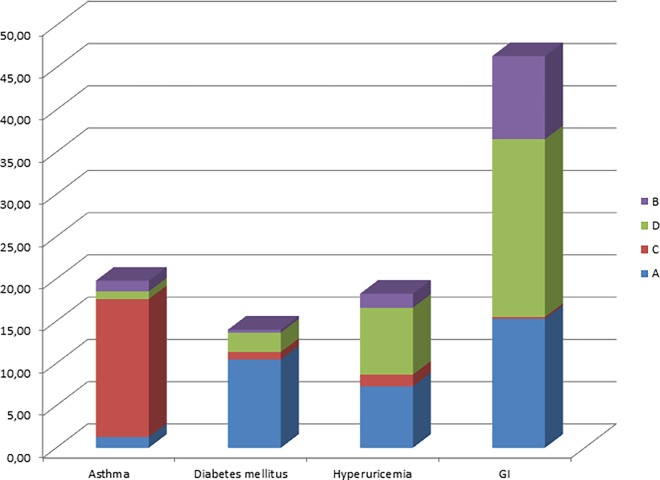
Examples of different distribution patterns of the concordance scores for the diseases asthma, diabetes, hyperuricemia and GI disorders. The values are percentages relative to the total number of patients (n = 2653). The blue part (A) represents the concordance between reported disease and specific medication, the red part (C) illustrates self-reports confirmed by non-specific medication. Green parts show the proportion of patients only reporting a disease without any suitable medication (D). The violet part (B) on top presents patients without the report of a disease but identified as likely having the disease due to the intake of a specific medication. The sum of A, C and D represents the prevalence according to self-reports (see Table 5). The distribution patterns vary widely among the different diseases.

Table 5 shows the distribution of scores for the 9 analyzed comorbidities and the combined cardiovascular disorder. The distribution varied between diseases and in particular the contributions of the scores A and D were very different. For selected diseases the pattern is illustrated in Fig 1 (for all diseases see S1 Fig).

**Table 5:** Distribution of concordance scores for different comorbidities (percentages based on the total number of included patients (n = 2653)).

Disease	Reported Prevalence (n)	Prevalence including B (n)	Concordance Scores (n)			
			A	B	C	D
Hypertension	56.1% (1489)	57.4% (1524)	548	35	804	137
Dyslipidemia	39.0% (1035)	43.8% (1163)	499	128	3	533
GI	36.5% (969)	46.4% (1230)	405	261	6	558
Mental disorders	21.4% (567)	24.9% (660)	213	93	28	326
Asthma	18.5% (491)	19.8% (525)	34	34	433	24
Hyperuricemia	16.6% (440)	18.2% (484)	193	44	37	210
Coronary heart disease	16.1% (426)	17.2% (455)	77	29	304	45
Osteoporosis	15.2% (402)	15.7% (417)	108	15	76	218
Diabetes mellitus	13.6% (362)	14.0% (371)	277	9	24	61
Combined CVD	60.8% (1612)	65.6% (1741)	1374	129	75	163

Scores A and B are based on disease-specific medication (ATC-Codes) while score C is assigned based on non-specific medication (ICD10-Codes). Scores A, C and D require the patient’s report of the respective disease. Only score B is based on specific medication only in the absence of a patient-reported diagnosis. Therefore scores A and C are directly comparable to each other whereas scores B and D are based on different sources of information.

The proportion of patients in whom the comorbidity was in accordance with specific medication was highest for diabetes mellitus. Only few patients were identified as taking diabetes-specific medication without reported diabetes (2.4% of 371 patients). Similarly, the proportion of patients with reported diabetes without specific or non-specific medication was low (16.4% of 371 patients).

In contrast to diabetes, reported asthma was associated with specific medication only in a minority of patients. This was a consequence of the fact that there are very few drugs on the market which are specific for asthma and not at the same time applicable in COPD. This reduces the potential to confirm asthma by medication in a cohort in which patients were required to have COPD. The percentage of patients reporting asthma without any medication indicating asthma was very low.

An intermediate pattern was observed for hyperuricemia. In a considerable proportion of patients the self-reported diagnosis was in accordance with the reported intake of specific medication, and the proportion of non-specific medication was low. On the other hand 47.7% of patients reported to have been diagnosed with hyperuricemia but took neither specific nor non-specific medication.

The analysis of coronary heart disease and hypertension showed a very high proportion of patients in whom the self-reported diagnosis was in accordance to non-specific medication. In contrast the percentage identified by specific medication was low. This reflected the fact that few drugs are available which are specifically targeted against one of these two diseases. This becomes apparent in the relatively low number in category A. Only a minority of patients with coronary heart disease or hypertension could be identified by specific drugs in the absence of a self-reported diagnosis. In comparison to the single diseases, the level of accordance between diagnosis and specific medication was much higher for the combined entity comprising hypertension, coronary heart disease and heart failure. This shows that the combined entity allowed for a greater number of specific medications (see S12 Table).

The patterns observed in the other diseases were similar to those of the diseases described above. Overall, about 51.5% of the reported comorbidities were confirmed by Approach I, without very large differences between diseases although the number of comorbidities analyzed was much higher than in Approach II. Despite the lower number of comorbidities analyzed, in Approach II the percentage confirmed by specific medication (concordance score A) showed even larger differences between diseases (Table 5 and S1 Fig).

**Fig 2:**
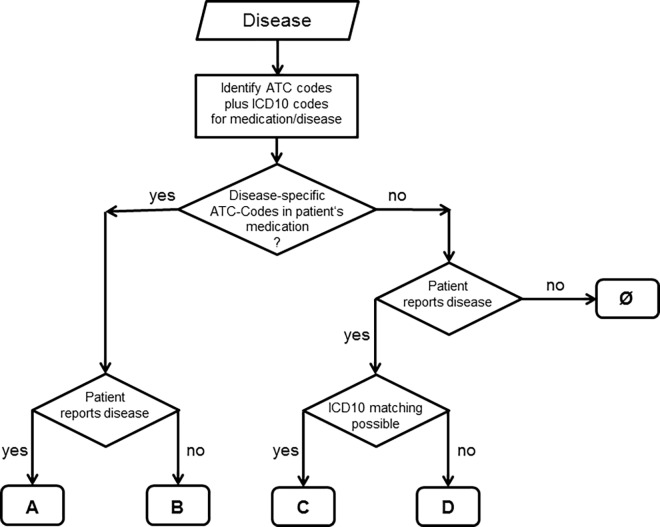
Diagram showing the logical structure of the combined categorization procedure (Approach I plus Approach II). A-D indicates the concordance scores, Ø the absence of the disease under study. ATC-Codes refer to the patients‘ medication, and ICD10 matching to the comparison of medication with the revised ICD10-Codes of the disease (for details see text).

The strategy comprising the combined Approaches I and II was equivalent to the flow chart shown in Fig 2. The structure of this chart also illustrates why it was possible to add the percentages of scores A-D as shown in Fig 1.

## Discussion

In COSYCONET, as in many other studies, patients were asked to report physician-diagnosed comorbidities (“Have you ever been diagnosed with…”). This type of assessment is required since it is practically impossible to verify a broad spectrum of comorbidities by clinical assessments at the study visits. Obviously, the information depends on the patients’ knowledge, memory and cooperation. It is also based on the sensitivity and specificity of the assessment of comorbidities during usual care by the patients’ physicians. Therefore it would be helpful to increase the reliability of patients’ reports with the help of other data available in the registry. For this purpose we developed a categorization system comprising four concordance scores for the reported diseases by using the information on medication. The major outcome was that about 50% of comorbidities were in accordance with medication when using the non-specific Approach I. For a selected number of important comorbidities Approach II showed that the degree of confirmation differed very much between diseases, particularly diabetes and asthma.

Many studies on multimorbidity have relied on questionnaires asking for self-reported doctor-diagnosed diseases [4, 22]. Studies on the relationship between self-reported and other medical data [5–7, 9–11] indicated sufficient reliability, especially for highly prevalent chronic conditions [6–9, 12], however the relationship differed between diseases. Using medical records as a gold standard [7, 9, 23] the sensitivities for different diseases turned out to be >0.7 indicating that self-reports can be a valid way to capture comorbidities despite the finding that for depression/anxiety and peptic ulcer disease sensitivities were lower [23]. These findings were explained by the fact that patients’ reports depend on disease severity, symptoms, the characteristics of the comorbidity and the need for ongoing monitoring [10]. Similar results were shown in a study that did not use a gold standard [12]; the concordance between self-reports and other sources of information was high for easily memorable, clearly defined conditions such as diabetes or heart disease, while it was lower for depression and lung disorders. Higher prevalences for self-reports compared to medical records might be due to patients’ recall bias or incomplete records [7, 24] but underreporting is also possible for diseases with low impact on daily symptoms [6].

In view of these findings the primary aim of our study was to evaluate to which extent information derived from medication can be used in different comorbidities; for this purpose we developed and described in detail a method utilizing these data.

Coelho et al. [14] already addressed the confirmation of self-reports by using medication, but no detailed information on the method was given. Computerized tools like the RxRisk Model [25] that have been used to relate ambulatory pharmacy data to chronic conditions cannot be used in all countries, since the pharmacy management differs and data may not be available in the proper format. In general, it seems that taking into account several sources of information for comorbidities leads to the best description of the patients’ comorbidity status [7, 13]. There is one study in patients with COPD which used medication (diabetes) or biomarkers (hypertension) to assess the prevalence of clinically relevant comorbidities, but no details were given on the procedure [26]. Biomarkers pose the problem that they might be affected by treatment. We therefore developed and describe such a procedure based on medication. As a further novel contribution concordance scores were defined providing different levels of confidence into the self-reported information on comorbidities.

In Approach I more than half of the reported diseases were found to be compatible with the intake of medication. This was encouraging when considering that some chronic conditions do not require regular medication and some may only have been present in the past. Nonetheless, this approach was limited in its confirmatory ability (concordance score C), since it did not account for the specificity of medication and all possible indications for a drug were admitted, e.g. in glucocorticoids more than 120.

For this reason Approach II was developed which allowed the addition of two further concordance scores (A and B). The basic feature was the use of disease-specific medications. If the patient’s report and the medication matched, the highest concordance score A was assigned. In analogy to the RxRisk model score B was defined as probable disease indicated by specific medication but without reporting of the disease by the patient. Although score B is to be considered lower than score A, the specificity of medication is strongly suggestive of the comorbidity. The lowest level of credibility (concordance score D) was given, when neither non-specific nor specific medication was identified although a patient reported a comorbidity. This is difficult to interpret since the comorbidity may have been cured or may be mild and does not require medication. In this case only more specific information, the comparison with the patients’ clinical state and/or biomarkers is capable of giving further clues.

The identification of specific drugs for GI disorders was difficult since many GI problems are treated not for chronic but acute conditions. Moreover acute problems of the GI tract are often caused by other conditions (e.g. diarrhea due to viral infection; vomiting due to use of cytostatic drugs). Therefore drugs for these types of symptoms were excluded (e.g. loperamide, metoclopramide). Another problem was that Over-the-counter (OTC) medication is available for the treatment of chronic GI problems (e.g. omeprazole). A prescribed medication supports the assumption that a physician diagnosed the self-reported comorbidity, whereas in case of OTC medications the self-reported disease must not be based on a physician’s diagnosis. Moreover, pantoprazole or omeprazole are often prescribed to prevent GI irritations caused by other drugs. This reduces the ability to infer a comorbidity from specific medication in this particular case.

The differentiation between asthma and other obstructive lung diseases via medication is a difficult issue. In COSYCONET this posed a particular problem due to the fact that all COSYCONET-patients were required to have a diagnosis of COPD. Many COPD medications are also prescribed in asthma, such as SABAs, LABAs or inhaled corticosteroids, and there are very few medications for asthma only. Therefore the opportunity for evaluation of asthma was low. Possibly a combined diagnosis “obstructive airway disorder” comprising asthma and COPD would result in a high degree of concordance.

Hypertension was the most often reported comorbidity in COSYCONET. Notably, many patients with hypertension are not categorized into the highest concordance score A since their hypertension therapeutics have many further indications. Coronary heart disease is also often treated by non-specific medications. We therefore combined coronary heart disease, hypertension and heart failure into a complex entity and used medication specific for one or more of these diseases to define concordance scores. This resulted in a high proportion of patients in concordance score A in comparison to the single diseases. In addition to the pharmaceutical argument given in the Methods section, we included the analysis of the combined group in order to illustrate that even with few, clinically heterogeneous diseases a remarkable gain in concordance score could be achieved, provided that there is medication specific for up to three of these diseases.

It will depend on the aim of the analyses regarding these comorbidities, whether a high degree of confirmation for a broader entity or a separation into single diseases is better. Due to this we cannot give a general recommendation how to use the scores. Self-reports are often considered sufficient in large epidemiological studies assessing prevalence estimates but score B may improve the estimates, for diseases on which most medication is specific.

In addition a detailed analysis of medication might be helpful for comparisons between patients merely reporting the disease versus those adequately treated. Information on specific medication might also be useful if this medication has pleiotropic effects extending beyond their specific indication [27, 28]. In contrast, patients with score D could be considered with caution in the analysis. This applies e.g. to the cardiovascular disorders which commonly require regular treatment. Overreporting as possibly reflected in score D might be based on patients’ misinterpretations of medical terminology [29]. We propose that by comparing different medication-based definitions of the disease it should be analyzed, whether exclusion of patients with score D creates more reliable groups. In addition, we expect that such comparisons could also be valuable in the analysis of quality of life data which might depend on the intake of medication in relation to disease severity. In our view, the ICD10-Code-based approach is useful for the quality check of data in studies relying on self-reports, since at least it can indicate compatibility between medication and report. These considerations illustrate that the different approaches and categories developed by us offer several options that can be chosen and adapted according to specific diseases and needs.

### Limitations

The main limitation of this study is the lack of a gold standard to which the data could be compared. Usually medical record abstraction is used as reference, but we could not assess diagnoses from the medical records of the patients. This underlines the potential value of a detailed medication history which might be the only information available in large studies. Even the use of historical data from prescription files could be useful if potential legal issues can be handled. Moreover, the distribution of scores varied between diseases, although their definition was the same. This limitation seems to be based in the characteristics of various diseases and difficult to avoid. A further limitation is that some diseases do not require regular medication or are curable and nevertheless may have been reported by the patient. There also may be conditions in which the disorder does not necessarily indicate a disease in the proper sense, but a side-effect of therapy or other diseases (e.g. GI).

Another reason for insufficient concordance between some diseases and prescribed medication might result from the type of questions for comorbidities. The COSYCONET study asked for any physician-based diagnosis in the patients’ lifetime. Especially for diseases strongly influenced by lifestyle (e.g. changes in nutrition or higher physical activity) or effectively cured (e.g. depression/anxiety in earlier life time) this can pose a problem which can be overcome by establishing a more detailed clinical history.

## Supporting Information

S1 COSYCONET Consortium Complete membership of the author group. (DOCX)

S1 Ethics Committees (DOCX)

S1 Fig Different distribution patterns of the concordance scores for all selected important comorbidities. The blue part (A) represents the concordance between reported disease and specific medication, the red part (C) illustrates self-reports confirmed by non-specific medication. Green parts show the proportion of patients only reporting a disease without any suitable medication (D). The violet part (B) on top presents patients without the report of a disease but identified as likely having the disease due to the intake of a specific medication. A, C and D shows the prevalence according to self-reports. The distribution patterns vary widely among the different diseases.
(TIF)

S1 Table ICD10 coding of all reported comorbidities as used in the analysis of Approach I. (DOCX)

S2 Table List of ICD10-Codes used for comparison of comorbidities and medication. (DOCX)

S3 Table Specific mediation and ATC-Codes for hypertension. (DOCX)

S4 Table Specific mediation and ATC-Codes for dyslipidemia. (DOCX)

S5 Table Specific mediation and ATC-Codes for GI disorders. (DOCX)

S6 Table Specific mediation and ATC-Codes for mental disorders. (DOCX)

S7 Table Specific mediation ATC-Codes for asthma. (DOCX)

S8 Table Specific mediation and ATC-Codes for hyperuricemia. (DOCX)

S9 Table Specific mediation and ATC-Codes for coronary heart disease. (DOCX)

S10 Table Specific mediation and ATC-Codes for osteoporosis. (DOCX)

S11 Table Specific mediation and ATC-Codes for diabetes mellitus. (DOCX)

S12 Table Specific mediation and ATC-Codes for the combined cardiovascular disorder. (DOCX)
