# Supplementary material for: Systematic Analysis of Self-Reported Comorbidities in Large Cohort Studies – A Novel Stepwise Approach by Evaluation of Medication
Source: PLoS One. 2016 Oct 28;11(10):e0163408. doi: 10.1371/journal.pone.0163408 (PMC5085029; doi:10.1371/journal.pone.0163408)
Supplement: S1 COSYCONET Consortium — (DOCX) [file pone.0163408.s001.docx]

| **Study Site** | **Principal Investigator** |
| --- | --- |
| **Philipps-Universität Marburg** | **Prof. Dr. Claus Vogelmeier** |
| Universitätsklinikum Marburg |  |
| Pneumologie |  |
| Baldingerstraße |  |
| 35043 Marburg |  |
| **Medizinische Hochschule Hannover** | **Prof. Dr. Tobias Welte** |
| Klinik für Pneumologie |  |
| Carl-Neuberg-Str. 1 |  |
| 30625 Hannover |  |
| **Medizinische Klinik/Campus Innenstadt** | **Prof. Dr. Huber** |
| Pneumologie |  |
| Ziemsenstr.1 |  |
| 80336 München |  |
| **Wissenschaftliches Institut Bethanien e.V.** | **Prof. Dr. Winfried J. Randerath** |
| Institut für Pneumologie an der Universität Witten/Herdecke |  |
| Aufderhöhe Straße 169-175 |  |
| 42699 Solingen |  |
| **Thoraxklinik-Heidelberg gGmbH** | **Prof. Dr. Felix Herth** |
| Pneumologie und Beatmungsmedizin |  |
| Amalienstr. 5 |  |
| 69126 Heidelberg |  |
|  |  |
| **Universität des Saarlandes** | **Prof. Dr. Dr. Robert Bals** |
| FR. 2.7 /Innere Medizin V |  |
| Abt. Pneumologie, Geb. 91 |  |
| Kirrberger Str. 100 |  |
| 66424 Homburg/Saar |  |
| **Universitätsmedizin Greifswald** | **Prof. Dr. Ralf Ewert** |
| Klinik und Poliklinik für Innere Medizin B |  |
| Pneumologie/Infektiologie, |  |
|  |  |
|  |  |
| **Kliniken Südostbayern AG** |  |
| Kreisklinik Bad Reichenhall | **Dr. Christian Geltner** |
| Pneumologie, Lungenzentrum SüdOst |  |
| Riedelstraße 5 |  |
| 83435 Bad Reichenhall |  |
| 20354 Hamburg | **Dr. Margret Jandl** |
|  |  |
|  |  |
| **Berufsgenossenschaftliches Universitätsklinikum** |  |
| **Bergmannsheil, Bochum** | **Prof. Dr. Andrea Koch** |
| Bürkle-de-la-Camp-Platz 1 | **Dr. Juliane Kronsbein** |
| 44789 Bochum |  |
| **Klinik Donaustauf** | **Prof. Dr. Michael Pfeifer** |
| Zentrum für Pneumologie |  |
| Ludwigstr. 68 |  |
| 93093 Donaustauf |  |
|  |  |
|  |  |
| **Fachkrankenhaus Coswig GmbH** | **Prof. Dr. Gerd Höffken** |
| Zentrum für Pneumologie, Thorax- und Gefäßchirurgie |  |
| Neucoswiger Str. 21 |  |
| 01640 Coswig |  |
|  |  |
| **Universitätsklinikum Heidelberg** | **Prof. Dr. Hugo A. Katus** |
| Medizinische Fakultät |  |
| Innere Medizin III |  |
| Im Nauenheimer Feld 672 |  |
| 69120 Heidelberg |  |
| **Fachklinik für Lungenerkrankungen Immenhausen** | **Prof. Dr. Stefan Andreas** |
| Pneumologische Lehrklinik der Universität Göttingen |  |
| Philippstiftung e. V. |  |
| Robert-Koch-Str. 3 |  |
| 34376 Immenhausen |  |
| **Universitätsklinikum Schleswig-Holstein** | **Prof. Dr. Burkhardt Bewig** |
| **Campus Kiel** |  |
| Klinik für Innere Medizin I |  |
| Arnold-Heller-Str. 3, Haus 6 |  |
| 24105 Kiel |  |
| **Forschungszentrum Borstel** | **Prof. Dr. Peter Zabel** |
| Leibnitz-Zentrum für Medizin und Biowissenschaften |  |
| Klinisches Studienzentrum |  |
| Parkallee 35 |  |
| 23845 Borstel |  |
| **Pneumologisches Forschungsinstitut** |  |
| **an der Lungenclinic Grosshansdorf GmbH** | **PD Dr. Henrik Watz** |
| Wöhrendamm 80 | **PD Dr. Anne-Marie Kirsten** |
| 22927 Großhansdorf |  |
|  |  |
| **Justus-Liebig-Universtität Gießen** | **Prof. Dr. Werner Seeger** |
| Zentrum für Innere Medizin |  |
| Medizinische Klinik und Poliklinik II |  |
| Klinikstr. 33 |  |
| 35392 Gießen |  |
| **Asklepios Klinik München-Gauting** |  |
| EvA-Study Center | **Prof. Dr. Jürgen Behr** |
| Hemholtz Zentrum München |  |
| Robert-Koch-Str. 29 |  |
| 82131 Gauting |  |
| **Klinikum Nürnberg Nord** | **Prof. Dr. Joachim Ficker** |
| KNN |  |
| Prof.-Ernst-Nathan-Str. 1 |  |
| 90419 Nürnberg |  |
| **Universitätsklinikum Ulm** |  |
| Studienzentrale Innere Medizin II/Pneumologie |  |
| Albert-Einstein-Str. 23 | **Dr. Cornelia Kropf-Sanchen** |
| 89081 Ulm |  |
|  |  |
| **Ev. Lungenklinik Berlin** | **Prof. Dr. Christian Grohé** |
| Lindenberger Weg 27 |  |
| Haus 205 |  |
| 13125 Berlin |  |
| **Universität Leipzig** | **Prof. Dr. Hubert Wirtz** |
| Medizinische Fakultät |  |
| Abt. Pneumologie |  |
| Liebigstr. 40 |  |
| 04103 Leipzig |  |
| **Missionsärztliche Klinik** | **Prof. Dr. Berthold H. Jany** |
| Gemeinnützige Gesellschaft mbH |  |
| Salvatorstr. 7 |  |
| 97074 Würzburg |  |
| **Schön Klinik** | **Prof. Dr. Klaus Kenn** |
| Berchtesgadener Land |  |
| Malterhöh 1 |  |
| 83471 Schönau am Königsee |  |
| **Krankenhaus Lindenbrunn, Coppenbrügge** | **Dr. Manfred Gogol** |
| VzBvS e.V. |  |
| Lindenbrunn 1 |  |
| 31863 Coppenbrügge |  |
| **Klinik Löwenstein gGmbH** | **Prof. Dr. Ulrich Wagner** |
| Geißhölzle 62 |  |
| 74245 Löwenstein |  |
| **Universitätsmedizin der** | **Prof. Dr. Roland Buhl** |
| **Johannes-Gutenberg-Universität Mainz** |  |
| Schwerpunkt Pneumologie |  |
| Gebäude 406, II OG |  |
| III. Medizinische Klinik |  |
| Langenbeckstr. 1 |  |
| 55131 Mainz |  |
| **Universitätsklinikum Rostock** | **Prof. Dr. J. Christian Virchow** |
| Zentrum für Innere Medizin |  |
| Abt. für Pneumologie |  |
| Ernst-Heydemann-Str. 6 |  |
| 18057 Rostock |  |
|  |  |
| **Ruhrlandklinik gGmbH Essen** | **Prof. Dr. H. Teschler** |
| Westdeutsches Lungenzentrum |  |
| Tüschener Weg 40 |  |
| 45239 Essen |  |
|  |  |
