# Supplementary material for: Systematic Analysis of Self-Reported Comorbidities in Large Cohort Studies – A Novel Stepwise Approach by Evaluation of Medication
Source: PLoS One. 2016 Oct 28;11(10):e0163408. doi: 10.1371/journal.pone.0163408 (PMC5085029; doi:10.1371/journal.pone.0163408)
Supplement: S1 Ethics Committees — (DOCX) [file pone.0163408.s002.docx]

**All local ethics comitees:**

(Bad Reichenhall (Ethikkommission bayerische Landesärztekammer); Berlin (Ethikkommission Ärztekammer Berlin); Bochum (Ethikkommission Medizinische Fakultät der RUB); Borstel (Ethikkommission Universität Lübeck); Coswig (Ethikkommission TU Dresden); Donaustauf (Ethikkommission Universitätsklinikum Regensburg); Essen (Ethikkommission Medizinische Fakultät Duisburg-Essen); Gießen (Ethikkommission Fachbereich Medizin); Greifswald (Ethikkommission Universitätsmedizin Greifswald); Großhansdorf (Ethikkommission Ärztekammer Schleswig-Holstein); Hamburg (Ethikkommission Ärztekammer Hamburg); MHH Hannover / Coppenbrügge (MHH Ethikkommission); Heidelberg Thorax/Uniklinik (Ethikkommission Universität Heidelberg); Homburg (Ethikkommission Saarbrücken); Immenhausen (Ethikkommission Landesärztekammer Hessen); Kiel (Ethikkommission Christian-Albrechts-Universität zu Kiel); Leipzig (Ethikkommission Universität Leipzig); Löwenstein (Ethikkommission Landesärztekammer Baden-Württemberg); Mainz (Ethikkommission Landesärztekammer Rheinland-Pfalz); München LMU/Gauting (Ethikkommission Klinikum Universität München); Nürnberg (Ethikkommission Friedrich-Alexander-Universität Erlangen Nürnberg); Rostock (Ethikkommission Universität Rostock); Berchtesgadener Land (Ethikkommission Land Salzburg); Schmallenberg (Ethikkommission Ärztekammer Westfalen-Lippe); Solingen (Ethikkommission Universität Witten-Herdecke); Ulm (Ethikkommission Universität Ulm); Würzburg (Ethikkommission Universität Würzburg))
