# Supplementary figures and images for: Systematic Analysis of Self-Reported Comorbidities in Large Cohort Studies – A Novel Stepwise Approach by Evaluation of Medication
Source: PLoS One. 2016 Oct 28;11(10):e0163408. doi: 10.1371/journal.pone.0163408 (PMC5085029; doi:10.1371/journal.pone.0163408)

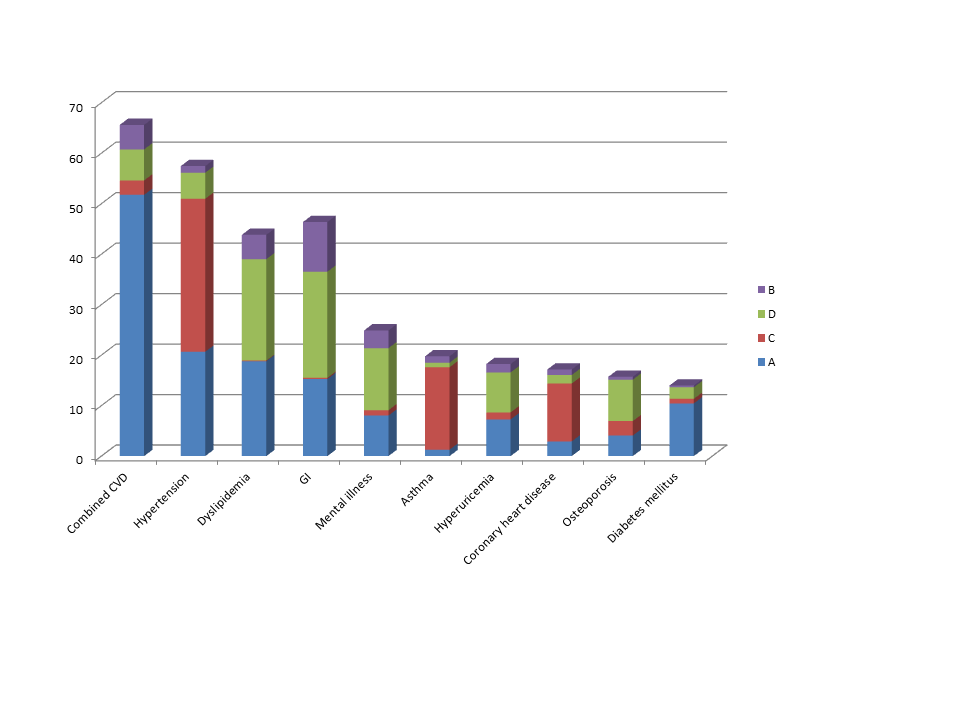

Supplement: S1 Fig — The blue part (A) represents the concordance between reported disease and specific medication, the red part (C) illustrates self-reports confirmed by non-specific medication. Green parts show the proportion of patients only reporting a disease without any suitable medication (D). The violet part (B) on top presents patients without the report of a disease but identified as likely having the disease due to the intake of a specific medication. A, C and D shows the prevalence according to self-reports. The distribution patterns vary widely among the different diseases. (TIF) [file pone.0163408.s003.tif]
