# Supplementary material for: Systematic Analysis of Self-Reported Comorbidities in Large Cohort Studies – A Novel Stepwise Approach by Evaluation of Medication
Source: PLoS One. 2016 Oct 28;11(10):e0163408. doi: 10.1371/journal.pone.0163408 (PMC5085029; doi:10.1371/journal.pone.0163408)
Supplement: S1 Table — (DOCX) [file pone.0163408.s004.docx]

**S1 Table: ICD10 coding of all reported comorbidities as used in the analysis of Approach I**

| Disease | ICD10-Code |
| --- | --- |
| Bronchiectasis | J47 |
| Pulmonary fibrosis | J84 |
| Sarcoidosis | D86 |
| Lung cancer | C34 |
| Sleep apnea | G47.3 |
| Hypertonie | I10-I15 |
| Coronary heart disease | I20; I24; I25 |
| Heart attack | I21-I22 |
| Cardiac arrhythmias | I47-I49 |
| Heart failure | I50 |
| Stroke | I63-I64 |
| Circulatory disorders of the brain | I65-I66; G45 |
| Circulatory problems in the legs | I73; I70.22 |
| Varicose veins , leg ulcers | I83; L97 |
| Vein thrombosis | I80; I82.9 |
| Bronchial asthma | J45-J46 |
| Chronic bronchitis | J41-J42 |
| COPD, emphysema | J43-J44 |
| Gastritis , gastroesophageal reflux , gastric, duodenal ulceration | K20-K31; R12 |
| Biliary diseases | K80-K87 |
| Liver cirrhosis, hepatitis | K70-K77; B15-B19 |
| Hypothyroidism | E00-E04 |
| Hyperthyroidism | E05 |
| Diabetes mellitus | E10-E14 |
| Dyslipidemia | E78 |
| Hyperuricemia | E79; M10 |
| Iron deficiency anemia | D50-D59 |
| Pyelonephritis | N10-N12 |
| Kidney stones , renal colic | N20-N23 |
| Cancer | C00-C33; C35-C97 |
| Osteoarthritis | M15-M19; M47 |
| Inflammatory joint diseases | M05-M09; M11-M14; M45 |
| Osteoporosis | M80-M85 |
| Migraine | G43 |
| Epilepsy | G40-G41 |
| Parkinson's disease | G20-G22 |
| Multiple sclerosis | G35 |
| Meningitis | G00-G03 |
| Mental disorders | F00; F07-F09;F20-F49; F51-F99 |
| Brain disorder | F01-F06; G31 |
| Polyneuropathy | G60-G64 |
| Eating Disorder | F50 |
| Alcohol addiction | F10-F19 |
| Drug addiction | F10-F19 |
| Hay fever | H10; J30 |
| Food allergy | T78 |
| Animal dander | J30 |
| Metal allergy | L23 |
| House dust allergy | J30 |
| Neurodermitis | L20 |
| Combined cardiovascular disorder | I10-I15; I20, I24, I25; I50 |
