# Supplementary material for: Systematic Analysis of Self-Reported Comorbidities in Large Cohort Studies – A Novel Stepwise Approach by Evaluation of Medication
Source: PLoS One. 2016 Oct 28;11(10):e0163408. doi: 10.1371/journal.pone.0163408 (PMC5085029; doi:10.1371/journal.pone.0163408)
Supplement: S2 Table — (DOCX) [file pone.0163408.s005.docx]

**S2 Table: List of ICD10-Codes used for comparison of comorbidities and medication**

| Disease | Disease ICD10-Code | Leading (three) digits of medication ICD10-Codes |
| --- | --- | --- |
| Bronchiectasis | J47 | J47.XX |
| Pulmonary fibrosis | J84 | J84.XX |
| Sarcoidosis | D86 | D86.XX |
| Lung cancer | C34 | C34.XX |
| Sleep apnea | G47.3 | G47.3X |
| Hypertonie | I10-I15 | I10.XX, I11.XX, I12.XX, I13.XX, I14.XX, I15.XX |
| Coronary heart disease | I20; I24; I25 | I20.XX, I24.XX, I25.XX |
| Heart attack | I21-I22 | I21.XX, I22.XX |
| Cardiac arrhythmias | I47-I49 | I47.XX, I48.XX, I49.XX |
| Heart failure | I50 | I50.XX |
| Stroke | I63-I64 | I63.XX, 64.XX |
| Circulatory disorders of the brain | I65-I66; G45 | I65.XX, I66.XX, G45.XX |
| Circulatory problems in the legs | I73; I70.22 | I73.XX, I70.22 |
| Varicose veins , leg ulcers | I83; L97 | I83.XX; L97.XX |
| Vein thrombosis | I80; I82.9 | I80.XX, I82.9X |
| Bronchial asthma | J45-J46 | J45.XX, J46.XX |
| Chronic bronchitis | J41-J42 | J41.XX, J42.XX |
| COPD, emphysema | J43-J44 | J43.XX, J44.XX |
| Gastritis , gastroesophageal reflux , gastric, duodenal ulcer | K20-K31; R12 | K20.XX, K21.XX to K31.XX, R12.XX |
| Biliary diseases | K80-K87 | K80.XX, K81.XX to K87.XX |
| Liver cirrhosis, hepatitis | K70-K77; B15-B19 | K70.XX, K71.XX to K77.XX, B15.XX to B19.XX |
| Hypothyroidism | E00-E04 | E00.XX, E01.XX, E02.XX, E03.XX, E04.XX |
| Hyperthyroidism | E05 | E05.XX |
| Diabetes mellitus | E10-E14 | E10.XX, E11.XX, E12.XX, E13.XX, E14.XX |
| Dyslipidemia | E78 | E78.XX |
| Gout | E79; M10 | E79.XX, M10.XX |
| Iron deficiency anemia | D50-D59 | D50.XX, D51.XX to D59.XX |
| Pyelonephritis | N10-N12 | N10.XX, N11.XX, N12.XX |
| Kidney stones , renal colic | N20-N23 | N20.XX, N21.XX; N22.XX, N23.XX |
| Cancer | C00-C33; C35-C97 | C00.XX to C33.XX, C35.XX to C97.XX |
| Osteoarthritis | M15-M19; M47 | M15.XX, M16.XX to M19.XX, M47.XX |
| Inflammatory joint diseases | M05-M09; M11-M14; M45 | M5.XX to M9.XX, M11.XX to M14.XX, M45.XX |
| Osteoporosis | M80-M85 | M80.XX to M85.XX |
| Migraine | G43 | G43.XX |
| Epilepsy | G40-G41 | G40.XX, G41.XX |
| Parkinson's disease | G20-G22 | G20.XX, G21.XX, G22.XX |
| Multiple sclerosis | G35 | G35.XX |
| Meningitis | G00-G03 | G00.XX, G01.XX, G02.XX, G03.XX |
| Mental illness | F00; F07-F09;F20-F49; F51-F99 | F00.XX, F07.XX, F08.XX, F09.XX, F21.XX to F49.XX, F51.XX to F99.XX |
| Brain disorder | F01-F06; G31 | F01.XX to F06.XX, G31.XX |
| Polyneuropathy | G60-G64 | G60.XX, G61.XX, G62.XX, G63.XX, G64.XX |
| Eating Disorder | F50 | F50.XX |
| Alcohol addiction | F10-F19 | F10.XX, F11.XX to F19.XX |
| Drug addiction | F10-F19 | F10.XX, F11.XX to F19.XX |
| Hay fever | H10; J30 | H10.XX, J30.XX |
| Food allergy | T78 | T78.XX |
| Animal dander | J30 | J30.XX |
| Metal allergy | L23 | L23.XX |
| House dust allergy | J30 | J30.XX |
| Neurodermitis | L20 | L20.XX |
| Combined cardiovascular disorder | I10-I15; I20, I24, I25; I50 | I10.XX to I15.XX; I20.XX or I24.XX or I25.XX; I50.XX |
