# Supplementary material for: Systematic Analysis of Self-Reported Comorbidities in Large Cohort Studies – A Novel Stepwise Approach by Evaluation of Medication
Source: PLoS One. 2016 Oct 28;11(10):e0163408. doi: 10.1371/journal.pone.0163408 (PMC5085029; doi:10.1371/journal.pone.0163408)
Supplement: S4 Table — (DOCX) [file pone.0163408.s007.docx]

S4 Table: Specific mediation and ATC-Codes for dyslipidemia

| ATC-Code | Drug |
| --- | --- |
| C10AA01 | Simvastatin |
| C10AA02 | Lovastatin |
| C10AA03 | Pravastatin |
| C10AA04 | Fluvastatin |
| C10AA05 | Atorvastatin |
| C10AA07 | Rosuvastatin |
| C10AB02 | Bezafibrat |
| C10AB04 | Gemfibrozil |
| C10AB05 | Fenofibrat |
| C10AC01 | Colestyramine |
| C10AC04 | Colesevelam |
| C10AD52 | Nicotinic acid, combinations |
| C10AX09 | Ezetimibe |
| C10BA02 | Simvastatin, ezetimibe |
