# Supplementary material for: Systematic Analysis of Self-Reported Comorbidities in Large Cohort Studies – A Novel Stepwise Approach by Evaluation of Medication
Source: PLoS One. 2016 Oct 28;11(10):e0163408. doi: 10.1371/journal.pone.0163408 (PMC5085029; doi:10.1371/journal.pone.0163408)
Supplement: S5 Table — (DOCX) [file pone.0163408.s008.docx]

S5 Table: Specific mediation and ATC-Codes for GI disorders

| ATC-Code | Drug |
| --- | --- |
| A02AB01 | Aluminium hydroxide |
| A02AB03 | Aluminium phosphate |
| A02AB04 | Dihydroxyaluminiumna triumcarbonate (Carbaldrat) |
| A02AC01 | Calcium carbonate |
| A02AD01 | Simple salt combinations |
| A02AD02 | Magaldrate |
| A02AD04 | Hydrotalcite |
| A02AD05 | Almasilate |
| A02AD10 | Aluminium oxide in combination with Magnesium hydroxide |
| A02BA01 | Cimetidine |
| A02BA02 | Ranitidine |
| A02BA03 | Famotidine |
| A02BC01 | Omeprazole |
| A02BC02 | Pantoprazole |
| A02BC03 | Lansoprazole |
| A02BC04 | Rabeprazole |
| A02BC05 | Esomeprazole |
| A02BC06 | Dexlansoprazole |
| A02BX02 | Sucralfate |
| A02BX03 | Pirenzepine |
| A02BX13 | Alginic acid |
| A02BX63 | Alginic acid, combinations |
| A03AA04 | Mebeverine |
| A07EC01 | Sulfasalazine |
| A07EC02 | Mesalazine |
| A07EC03 | Olsalazine |
| A03AP30 | Combinations |
