# Supplementary material for: Systematic Analysis of Self-Reported Comorbidities in Large Cohort Studies – A Novel Stepwise Approach by Evaluation of Medication
Source: PLoS One. 2016 Oct 28;11(10):e0163408. doi: 10.1371/journal.pone.0163408 (PMC5085029; doi:10.1371/journal.pone.0163408)
Supplement: S6 Table — (DOCX) [file pone.0163408.s009.docx]

S6 Table: Specific mediation and ATC-Codes for mental disorders

| ATC-Code | Drug |
| --- | --- |
| N05AA05 | Triflupromazine |
| N05AB02 | Fluphenazine |
| N05AB03 | Perphenazine |
| N05AB10 | Perazine |
| N05AC02 | Thioridazine |
| N05AD01 | Haloperidol |
| N05AD03 | Melperone |
| N05AD05 | Pipamperone |
| N05AD06 | Bromperidol |
| N05AD07 | Benperidol |
| N05AD08 | Droperidol |
| N05AE03 | Sertindol |
| N05AE04 | Ziprasidone |
| N05AE05 | Lurasidon |
| N05AF01 | Flupentixol |
| N05AF03 | Chlorprothixene |
| N05AF05 | Zuclopenthixol |
| N05AG01 | Fluspirilene |
| N05AG02 | Pimozide |
| N05AH01 | Loxapine |
| N05AH02 | Clozapine |
| N05AH03 | Olanzapine |
| N05AH04 | Quetiapine |
| N05AH05 | Asenapine |
| N05AL01 | Sulpiride |
| N05AL05 | Amisulpride |
| N05AN01 | Lithium |
| N05AX07 | Prothipendyl |
| N05AX08 | Risperidone |
| N05AX12 | Aripiprazole |
| N05AX13 | Paliperidone |
| N05BA01 | Diazepam |
| N05BA02 | Chlordiazepoxide |
| N05BA03 | Medazepam |
| N05BA04 | Oxazepam |
| N05BA05 | Chlorazepate |
| N05BA06 | Lorazepam |
| N05BA08 | Bromazepam |
| N05BA09 | Clobazam |
| N05BA11 | Prazepam |
| N05BA12 | Alprazolam |
| N05BE01 | Buspirone |
| N06AA05 | Opipramol |
| N06AA06 | Trimipramine |
| N06AA10 | Nortriptyline |
| N06AA12 | Doxepin |
| N06AA16 | Dosulepin |
| N06AA21 | Maprotiline |
| N06AA25 | Amitriptylinoxide |
| N06AB03 | Fluoxetine |
| N06AB04 | Citalopram |
| N06AB05 | Paroxetine |
| N06AB06 | Sertraline |
| N06AB08 | Fluvoxamine |
| N06AB10 | Escitalopram |
| N06CA02 | Melitracen and psycholeptics |
| N06AF04 | Tranylcypromine |
| N06AG02 | Moclobemide |
| N06AX03 | Mianserin |
| N06AX05 | Trazodone |
| N06AX11 | Mirtazapine |
| N06AX12 | Bupropion |
| N06AX14 | Tianeptine |
| N06AX16 | Venlafaxine |
| N06AX18 | Reboxetine |
| N06AX21 | Duloxetine |
| N06AX22 | Agomelatine |
| N06AX26 | Vortioxetine |
