# Supplementary material for: Systematic Analysis of Self-Reported Comorbidities in Large Cohort Studies – A Novel Stepwise Approach by Evaluation of Medication
Source: PLoS One. 2016 Oct 28;11(10):e0163408. doi: 10.1371/journal.pone.0163408 (PMC5085029; doi:10.1371/journal.pone.0163408)
Supplement: S7 Table — (DOCX) [file pone.0163408.s010.docx]

S7 Table: Specific mediation ATC-Codes for asthma

| ATC-Code | Drug |
| --- | --- |
| R03BC01 | Cromolyn |
| R03AK05 | Reproterol and cromolyn, disodium salt |
| R03DC03 | Montelukast |
| R03DX05 | Omalizumab |
