# Supplementary material for: Systematic Analysis of Self-Reported Comorbidities in Large Cohort Studies – A Novel Stepwise Approach by Evaluation of Medication
Source: PLoS One. 2016 Oct 28;11(10):e0163408. doi: 10.1371/journal.pone.0163408 (PMC5085029; doi:10.1371/journal.pone.0163408)
Supplement: S8 Table — (DOCX) [file pone.0163408.s011.docx]

S8 Table: Specific mediation and ATC-Codes for hyperuricemia

| ATC-Code | Drug |
| --- | --- |
| M04AA01 | Allopurinol |
| M04AA03 | Febuxostat |
| M04AA51 | Allopurinol, combinations |
| M04AB01 | Probenecid |
| M04AB03 | Benzbromarone |
| M04AC01 | Colchicine |
