# Supplementary material for: Systematic Analysis of Self-Reported Comorbidities in Large Cohort Studies – A Novel Stepwise Approach by Evaluation of Medication
Source: PLoS One. 2016 Oct 28;11(10):e0163408. doi: 10.1371/journal.pone.0163408 (PMC5085029; doi:10.1371/journal.pone.0163408)
Supplement: S9 Table — (DOCX) [file pone.0163408.s012.docx]

S9 Table: Specific mediation and ATC-Codes for coronary heart disease

| ATC-Code | Drug |
| --- | --- |
| C01DA02 | Glyceryl trinitrate |
| C01DA05 | Nitropenta |
| C01DA08 | Isosorbide dinitrate |
| C01DA14 | Isosorbid mononitrate |
| C01DX11 | Trapidil |
| C01DX12 | Molsidomin |
| C01EB18 | Ranolazine |
| C01DX16 | Nicorandil |
