# Supplementary material for: Systematic Analysis of Self-Reported Comorbidities in Large Cohort Studies – A Novel Stepwise Approach by Evaluation of Medication
Source: PLoS One. 2016 Oct 28;11(10):e0163408. doi: 10.1371/journal.pone.0163408 (PMC5085029; doi:10.1371/journal.pone.0163408)
Supplement: S10 Table — (DOCX) [file pone.0163408.s013.docx]

S10 Table: Specific mediation and ATC-Codes for osteoporosis

| ATC-Code | Drug |
| --- | --- |
| M05BA01 | Etidronic acid |
| M05BA02 | Clodronic acid |
| M05BA03 | Pamidronic acid |
| M05BA04 | Alendronic acid |
| M05BA05 | Tiludronic acid |
| M05BA06 | Ibandronic acid |
| M05BA07 | Risedronic acid |
| M05BA08 | Zoledronic acid |
| M05BB01 | Etidronic acid and calcium, sequential preparation |
| M05BB02 | Risedronic acid and calcium, sequential preparation |
| M05BB03 | Alendronic acid and colecalciferol |
| M05BB04 | Risedronic acid, calcium and colecalciferol, sequential preparation |
| M05BB05 | Alendronsäure, calcium and colecalciferol, sequential preparation |
| M05BB06 | Alendronic acid and alfacalcidol, sequential preparation |
| M05BB07 | Risedronic acid and colecalciferol |
| M05BB08 | Ibandronic acid and colecalciferol |
| M05BB09 | Ibandronic acid and calcium |
| M05BB10 | Ibandronic acid, calcium and colecalciferol, sequential preparation |
| M05BX03 | Strontium ranelate |
| M05BX04 | Denosumab |
| G03XC01 | Raloxifene |
| G03XC02 | Bazedoxifene |
