# Supplementary material for: Systematic Analysis of Self-Reported Comorbidities in Large Cohort Studies – A Novel Stepwise Approach by Evaluation of Medication
Source: PLoS One. 2016 Oct 28;11(10):e0163408. doi: 10.1371/journal.pone.0163408 (PMC5085029; doi:10.1371/journal.pone.0163408)
Supplement: S11 Table — (DOCX) [file pone.0163408.s014.docx]

S11 Table: Specific mediation and ATC codes for diabetes mellitus

| ATC-Code | Drug |
| --- | --- |
| A10AB01 | Insulin (human) |
| A10AB04 | Insulin lispro |
| A10AB05 | Insulin aspart |
| A10AB06 | Insulin glulisin |
| A10AC01 | Insulin (human) |
| A10AC04 | Insulin lispro |
| A10AD01 | Insulin (human) |
| A10AD04 | Insulin lispro |
| A10AD05 | Insulin aspart |
| A10AE04 | Insulin glargin |
| A10AE05 | Insulin detemir |
| A10AE06 | Insulin degludec |
| A10AE56 | Insulin degludec und Liraglutid |
| A10BA02 | Metformin |
| A10BB01 | Glibenclamide |
| A10BB08 | Gliquidone |
| A10BB09 | Gliclazide |
| A10BB12 | Glimepiride |
| A10BD05 | Metformin and pioglitazone |
| A10BD07 | Metformin and sitagliptin |
| A10BD08 | Metformin and vildagliptin |
| A10BD10 | Metformin and saxagliptin |
| A10BD15 | Metformin and dapagliflozin |
| A10BD19 | Metformin and glibenclamide |
| A10BF01 | Acarbose |
| A10BF02 | Miglitol |
| A10BG03 | Pioglitazone |
| A10BH01 | Sitagliptin |
| A10BH02 | Vildagliptin |
| A10BH03 | Saxagliptin |
| A10BX02 | Repaglinid |
| A10BX03 | Nateglinid |
| A10BX04 | Exenatid |
| A10BX07 | Liraglutide |
| A10BX09 | Dapagliflozin |
| A10BX10 | Lixisenatide |
| A10BX11 | Canagliflozin |
| A10BX12 | Empagliflozin |
