# Supplementary material for: Systematic Analysis of Self-Reported Comorbidities in Large Cohort Studies – A Novel Stepwise Approach by Evaluation of Medication
Source: PLoS One. 2016 Oct 28;11(10):e0163408. doi: 10.1371/journal.pone.0163408 (PMC5085029; doi:10.1371/journal.pone.0163408)
Supplement: S12 Table — (DOCX) [file pone.0163408.s015.docx]

S12 Table: Specific mediation and ATC-Codes for the combined cardiovascular disorder

| ATC-Code | Drug |
| --- | --- |
| C01DA02 | Glyceryl trinitrate |
| C01DA05 | Nitropenta |
| C01DA08 | Isosorbide dinitrate |
| C01DA14 | Isosorbid mononitrate |
| C01DX11 | Trapidil |
| C01DX12 | Molsidomin |
| C01DX16 | Nicorandil |
| C01EB17 | Ivabradine |
| C01EB18 | Ranolazine |
| C02AB01 | Methyldopa |
| C02AC01 | Clonidine |
| C02AC05 | Moxonidine |
| C02CA06 | Uradipil |
| C02CA08 | Terazosin |
| C02DB01 | Dihydralazine |
| C02DC01 | Minoxidil |
| C07AA03 | Pindolol |
| C07AB02 | Metoprolol |
| C07AB03 | Atenolol |
| C07AB04 | Acebutolol |
| C07AB05 | Betaxolol |
| C07AB07 | Tooprolol |
| C07AB08 | Celiprolol |
| C07AB12 | Nebivolol |
| C07AB13 | Talinolol |
| C07AG02 | Carvedilol |
| C07BA05 | Propranolol and thiazide |
| C07BB22 | Metoprolol and hydrochlorothiazide |
| C07BB27 | Bisoprolol and hydrochlorothioazide |
| C07CA51 | Penbutolol and furosemide |
| C07CA52 | Penbutolol and piratanide |
| C07CB02 | Metoprolol and other diuretics |
| C07CB03 | Atenolol and other diuretics |
| C07CB53 | Atenolol and other diuretics, combinations |
| C07DA06 | Timolol, thiazide and other diuretics |
| C07DA25 | Propranolol, hydrochlorothiazide and triamteren |
| C07FB23 | Atenolol and Nifedipine |
| C07FB24 | Metoprolol and Felodipine |
| C08CA01 | Amlodipine |
| C08CA02 | Felodipine |
| C08CA03 | Isradipine |
| C08CA05 | Nifedipine |
| C08CA07 | Nisoldipine |
| C08CA08 | Nitrendipine |
| C08CA10 | Nilvadipine |
| C08CA11 | Manidipine |
| C08CA13 | Lercanidipine |
| C08DB01 | Diltiazem |
| C08GA01 | Nifedipine and diuretics |
| C08GA23 | Verapamil and hydrochlorothiazide |
| C08GA53 | Verapamil, hydrochlorothiazide and triamteren |
| C09AA01 | Captopril |
| C09AA02 | Enalapril |
| C09AA03 | Lisinopril |
| C09AA04 | Perindopril |
| C09AA05 | Ramipril |
| C09AA06 | Quinapril |
| C09AA07 | Benazepril |
| C09AA08 | Cilazapril |
| C09AA09 | Fosinopril |
| C09AA10 | Trandolapril |
| C09AA11 | Spirapril |
| C09AA13 | Moexipril |
| C09AA15 | Zofenopril |
| C09BA21 | Captopril and hydrochlorothiazide |
| C09BA22 | Enalapril and hydrochlorothiazide |
| C09BA23 | Lisinopril and hydrochlorothiazide |
| C09BA25 | Ramipril and hydrochlorothiazide |
| C09BA26 | Quinapril and hydrochlorothiazide |
| C09BA27 | Benazepril and hydrochlorothiazide |
| C09BA28 | Cilazapril and hydrochlorothiazide |
| C09BA29 | Fosinapril and hydrochlorothiazide |
| C09BA33 | Moexipril and hydrochlorothiazide |
| C09BA35 | Zofenopril and hydrochlorothiazide |
| C09BA54 | Perindopril and indapamide |
| C09BA55 | Ramipril and piretanide |
| C09BB02 | Enalapril and lercanidipine |
| C09BB04 | Perindopril and amlodipine |
| C09BB05 | Ramipril and felodipine |
| C09BB06 | Enalapril and nitrendipine |
| C09BB07 | Ramipril and amlodipine |
| C09BB10 | Trandolapril and verapamil |
| C09BB12 | Delapril and manidipine |
| C09CA01 | Losartan |
| C09CA02 | Eprosartan |
| C09CA03 | Valsartan |
| C09CA04 | Irbesartan |
| C09CA06 | Candesartan |
| C09CA07 | Telmisartan |
| C09CA08 | Olmesartan medoxomil |
| C09CA09 | Azilsartan medoxomil |
| C09DA21 | Losartan and hydrochlorothiazide |
| C09DA22 | Eprosartan and hydrochlorothiazide |
| C09DA23 | Valsartan and hydrochlorothiazide |
| C09DA24 | Irbesartan and hydrochlorothiazide |
| C09DA26 | Candesartan and hydrochlorothiazide |
| C09DA27 | Temisartan and hydrochlorothiazide |
| C09DA28 | Olmesartan medoxomil and hydrochlorothiazide |
| C09DB01 | Valsartan and amlodipine |
| C09DB02 | Olmesartan medoxomil and amlodipine |
| C09DB04 | Telmisartan and amlodipine |
| C09DB05 | Irbesartan and amlodipine |
| C09DB07 | Candesartan and amlodipine |
| C09DX01 | Valsartan, amlodipine and hydrochlorothiazide |
| C09DX03 | Olmesartan medoxomil, amlodipin and hydrochlorothiazide |
| C09XA02 | Aliskiren |
| C09XA52 | Aliskiren and hydrochlorothiazide |
